# Supplementary material for: Systematics of putative euparkeriids (Diapsida: Archosauriformes) from the Triassic of China
Source: PeerJ. 2014 Nov 25;2:e658. doi: 10.7717/peerj.658 (PMC4250070; doi:10.7717/peerj.658)
Supplement: Table S1 — Measurements holotypes of Halazhaisuchus qiaoensis and ‘Turfanosuchus shageduensis’ and of holotype and paratypes of ‘Wangisuchus tzeyii’. [file peerj-02-658-s001.docx]

Table S1. **Supplementary table of measurements.** Measurements of the holotypes of *Halazhaisuchus qiaoensis* and “*Turfanosuchus shageduensis*” and of holotype and paratypes of “*Wangisuchus tzeyii*”

a.p.=as preserved

est.=estimate

| **Description of measurement** | **Measurement (mm)** |
| --- | --- |
| ***Halazhaisuchus qiaoensis*** |  |
| **IVPP V6027-1** | |
| **Cervical and dorsal vertebrae** | |
| ***First preserved cervical vertebra*** |  |
| Length of centrum | 21.9 |
| Height of centrum anteriorly | 17.6 |
| Height of centrum posteriorly | 16.4 |
| Width of centrum anteriorly | 14.1 |
| Width of centrum posteriorly | 14.7 |
| Minimum width of centrum | 7.6 |
| Minimum height of centrum | 13.4 |
| Distance across prezygapophyses | 16.7 a.p. |
| Distance across postzygapophyses | 19.7 |
| Length from anterior end of pre- to posterior end of postzygapophysis | 26.2 a.p. |
| Height of neural spine from base | 11.1 a.p. |
| Length of neural spine at tip | 14.1 a.p. |
| Width of neural spine at tip | 5.9 a.p. |
| Length of neural spine at base | 12 |
| Width of neural spine at base | 5.4 |
| Width of right diapophysis at base | 8.76 |
| Height of right diapophysis at base | 5.7 |
| Width of right diapophysis at tip | 7.4 |
| Height of right diapophysis at tip | 5 |
| Width of right parapophysis | 7.9 |
| Height of right parapophysis | 5.2 |
| Width of left diapophysis at base | 7.8 |
| Height of left diapophysis at base | 4.8 |
| Width of left diapophysis at tip | 7.2 a.p. |
| Height of left diapophysis at tip | 3.9 a.p. |
| Width of left parapophysis | 5.6 est. |
| Height of left parapophysis | 5.6 |
| ***Second preserved cervical vertebra*** |  |
| Length of centrum | 22.1 |
| Height of centrum anteriorly | 17.8 |
| Height of centrum posteriorly | 15 |
| Width of centrum anteriorly | 16.4 |
| Width of centrum posteriorly | 14.5 |
| Width of centrum at narrowest | 6.6 |
| Height of centrum at shortest | 10.8 |
| Distance across prezgapophyses | 22.7 |
| Distance across postzygapophyses | 19.5 |
| Length between anterior of pre- and posterior of postzygapophyses | 27.6 |
| Height of neural spine from base | 15.8 |
| Length of neural spine at tip | 19.4 |
| Width of neural spine at tip | 11.9 |
| Length of neural spine at base | 11.8 |
| Width of neural spine at base | 5.2 |
| Width of right diapophysis at base | 6.5 |
| Height of right diapophysis at base | 5.2 |
| Width of right diapophysis at tip | 6.4 |
| Height of right diapophysis at tip | 7 |
| Width of right parapophysis | 5.5 |
| Height of right parapophysis | 6.6 |
| Width of left diapophysis at base | 5.7 |
| Height of left diapophysis at base | 6.3 |
| Width of left diapophysis at tip | 4.1 a.p. |
| Height of left diapophysis at tip | 5.22 a.p. |
| Width of left parapophysis | 6.1 est. |
| Third from anteriormost cervical |  |
| Length between anterior of pre- and posterior of postzygapophyses | 24.4 |
| Length of neural spine at tip | 17.4 |
| Width of neural spine at tip | 12.8 |
| Length of neural spine at base | 11.1 |
| Width of neural spine at base | 6.4 |
| ***First preserved dorsal vertebra*** |  |
| Length of centrum | 9.2 a.p. |
| Height of centrum posteriorly | 16.2 |
| Width of centrum posteriorly | 17 |
| Width of centrum at narrowest | 9.6 a.p. |
| Height of centrum at shortest | 9.6 a.p. |
| Distance across prezgapophyses | 19.1 est. |
| Distance across postzygapophyses | 17.3 |
| Length between anterior of pre- and posterior of postzygapophyses | 22.3 |
| Length of neural spine at tip | 15.6 |
| Width of neural spine at tip | 10.8 |
| Length of neural spine at base | 9.43 |
| Width of neural spine at base | 6.8 |
| Width of right diapophysis at base | 7.9 |
| Height of right diapophysis at base | 5 |
| Width of right diapophysis at tip | 9 |
| Height of right diapophysis at tip | 7.7 |
| Width of right parapophysis | 6.4 est. |
| Height of right parapophysis | 5.5 est. |
| ***Second preserved dorsal vertebra*** |  |
| Length of centrum | 21.8 |
| Height of centrum anteriorly | 20.1 |
| Height of centrum posteriorly | 17.7 |
| Width of centrum anteriorly | 20 est. |
| Width of centrum posteriorly | 15.7 |
| Width of centrum at narrowest | 7.4 |
| Height of centrum at shortest | 11.4 |
| Distance across prezgapophyses | 18.1 |
| Length between anterior of pre- and posterior of postzygapophyses | 25 |
| Length of neural spine at tip | 19.2 |
| Width of neural spine at tip | 11.5 |
| Length of neural spine at base | 10.9 |
| Width of neural spine at base | 5.5 |
| Width of right diapophysis at base | 7.4 |
| Height of right diapophysis at base | 5.5 |
| Width of right diapophysis at tip | 8.13 |
| Height of right diapophysis at tip | 8.7 |
| Width of right parapophysis | 5.6 |
| Height of right parapophysis | 9.2 |
| Width of left diapophysis at base | 4.1 est. |
| Height of left diapophysis at base | 4.1 est. |
| ***Third preserved dorsal vertebra*** |  |
| Length of centrum | 14.1 a.p. |
| Height of centrum anteriorly | 16.5 |
| Height of centrum posteriorly |  |
| Width of centrum anteriorly | 14.1 |
| Width of centrum at narrowest | 6.7 a.p. |
| Height of centrum at shortest | 14 a.p. |
| Length of neural spine at tip | 20 |
| Width of neural spine at tip | 13.3 |
| Length of neural spine at base | 6.6 a.p. |
| Width of neural spine at base | 5.7 a.p. |
| Width of right diapophysis at base | 7.4 |
| Height of right diapophysis at base | 5.5 |
| Width of right diapophysis at tip | 6.6 |
| Height of right diapophysis at tip | 4.5 |
| Width of right parapophysis | 5.9 |
| Height of right parapophysis | 7.3 |
| Width of left diapophysis at base | 10.3 est. |
| Height of left diapophysis at base | 6.2 est. |
| Width of left parapophysis | 5.9 |
| Height of left parapophysis | 5.9 |
| ***More anterior osteoderm on left side above second preserved dorsal vertebra*** |  |
| Maximum length | 25.2 |
| Maximum width | 12.4 |
| Maximum depth | 4.2 |
| IVPP V6027-2 |  |
| **Dorsal vertebrae** |  |
| ***First preserved dorsal vertebra*** | |
| Length of centrum | 19.7 |
| Height of centrum anteriorly | 16.3 |
| Height of centrum posteriorly | 16 |
| Width of centrum anteriorly | 16.3 |
| Width of centrum posteriorly | 16.6 |
| Width of centrum at narrowest | 8.6 |
| Height of centrum at shortest | 6.8 |
| Distance across postzygapophyses | 14.5 a.p. |
| Length between anterior of pre- and posterior of postzygapophyses | 28.9 |
| Length of neural spine at base | 14.1 |
| Width of neural spine at base | 4 |
| ***Second preserved dorsal vertebra*** |  |
| Length of centrum | 22.3 |
| Height of centrum anteriorly | 16.8 |
| Height of centrum posteriorly | 16.1 |
| Width of centrum anteriorly | 17.6 |
| Width of centrum posteriorly | 15.3 |
| Width of centrum at narrowest | 7.4 |
| Height of centrum at shortest | 7.6 |
| Distance across prezgapophyses | 14.6 a.p. |
| Distance across postzygapophyses | 16.2 |
| Length between anterior of pre- and posterior of postzygapophyses | 30.3 |
| Height of neural spine from base | 16.2 |
| Length of neural spine at tip | 20.8 |
| Width of neural spine at tip | 7.4 |
| Length of neural spine at base | 13.2 |
| Width of neural spine at base | 4.2 |
| Width of right diapophysis at base | 6.8 a.p. |
| Height of right diapophysis at base | 5.2 a.p. |
| Width of right parapophysis | 3.8 |
| Height of right parapophysis | 5.1 |
| Width of left diapophysis at base | 5.4 est. |
| Height of left diapophysis at base | 7.5 est. |
| Width of left parapophysis | 4.5 est. |
| Height of left parapophysis | 6.0 est. |
| ***Third preserved dorsal vertebra*** |  |
| Length of centrum | 19.7 |
| Height of centrum anteriorly | 14.1 |
| Height of centrum posteriorly | 15.9 |
| Width of centrum anteriorly | 14.5 |
| Width of centrum posteriorly | 16 |
| Width of centrum at narrowest | 7.7 |
| Height of centrum at shortest | 6.9 |
| Distance across prezgapophyses | 17.2 |
| Distance across postzygapophyses | 1.5 a.p. |
| Length between anterior of pre- and posterior of postzygapophyses | 18.6 a.p. |
| Height of neural spine from base | 18.3 |
| Length of neural spine at tip | 21.1 |
| Width of neural spine at tip | 6.8 |
| Length of neural spine at base | 8.8 |
| Width of neural spine at base | 4.7 |
| Width of right transverse process at base | 9.8 |
| Height of right transverse process at base | 7.7 |
| Width of right transverse process at tip | 8.9 a.p. |
| Height of right transverse process at tip | 7.8 a.p. |
| Width of left transverse process at base | 8.9 |
| Height of left transverse process at base | 8.1 |
| Width of left transverse process at tip | 8.3 |
| Height of left transverse process at tip | 7.1 |
| ***Fourth preserved dorsal vertebra*** |  |
| Length of centrum | 20.5 |
| Height of centrum anteriorly | 16 |
| Height of centrum posteriorly | 17.4 |
| Width of centrum anteriorly | 16.1 |
| Width of centrum posteriorly | 17.1 |
| Width of centrum at narrowest | 8.3 |
| Height of centrum at shortest | 8.9 |
| Distance across prezgapophyses | 16.9 |
| Distance across postzygapophyses | 19.1 |
| Length between anterior of pre- and posterior of postzygapophyses | 27.1 |
| Height of neural spine from base | 16.9 |
| Length of neural spine at tip | 22.3 |
| Width of neural spine at tip | 6.5 |
| Length of neural spine at base | 14.5 |
| Width of neural spine at base | 5.4 |
| Width of right transverse process at base | 8.4 |
| Height of right transverse process at base | 6.7 |
| Width of right transverse process at tip | 7.1 |
| Height of right transverse process at tip | 6.3 |
| Width of left transverse process at base | 8.7 |
| Height of left transverse process at base | 8.8 |
| Width of left transverse process at tip | 7.5 |
| Height of left transverse process at tip | 8.9 |
| ***Fifith preserved dorsal vertebra*** |  |
| Length of centrum | 18.8 |
| Height of centrum anteriorly | 17.2 |
| Height of centrum posteriorly | 17.5 |
| Width of centrum anteriorly | 16.3 |
| Width of centrum posteriorly | 17.9 |
| Width of centrum at narrowest | 8.2 |
| Height of centrum at shortest | 9.5 |
| Distance across prezgapophyses | 19.3 |
| Length between anterior of pre- and posterior of postzygapophyses | 28.1 |
| Height of neural spine from base | 16.5 |
| Length of neural spine at tip | 19.7 |
| Width of neural spine at tip | 5.7 |
| Length of neural spine at base | 12.7 est. |
| Width of neural spine at base | 5.9 |
| Width of right transverse process at base | 8.2 |
| Height of right transverse process at base | 6.5 |
| Width of right transverse process at tip | 7.4 |
| Height of right transverse process at tip | 6.2 |
| Width of left transverse process at base | 7.7 |
| Height of left transverse process at base | 6.2 |
| Width of left transverse process at tip | 7.5 |
| Height of left transverse process at tip | 5.9 |
| ***Sixth preserved dorsal vertebra*** |  |
| Length of centrum | 18.2 |
| Height of centrum anteriorly | 16.2 |
| Height of centrum posteriorly | 14.8 |
| Width of centrum anteriorly | 16.8 |
| Width of centrum posteriorly | 17.4 |
| Width of centrum at narrowest | 8.4 |
| Height of centrum at shortest | 7.3 |
| Length between anterior of pre- and posterior of postzygapophyses | 19.6 est. |
| Height of neural spine from base | 15.9 |
| Length of neural spine at tip | 19.7 |
| Width of neural spine at tip | 7.5 |
| Length of neural spine at base | 12.1 |
| Width of neural spine at base | 4.3 |
| Width of right transverse process at base | 8.7 |
| Height of right transverse process at base | 6.7 |
| Width of right transverse process at tip | 5.3 |
| Height of right transverse process at tip | 5.1 |
| Width of left transverse process at tip |  |
| Height of left transverse process at tip |  |
| ***Seventh preserved dorsal vertebra*** |  |
| Length of centrum | 17.5 |
| Height of centrum anteriorly | 15.5 a.p. |
| Height of centrum posteriorly | 18.8 |
| Width of centrum anteriorly | 16.6 |
| Width of centrum posteriorly | 18.4 |
| Width of centrum at narrowest | 9.7 |
| Height of centrum at shortest | 8.2 a.p. |
| ***More anterior osteoderm above fifth preserved dorsal vertebra preserved*** | |
| Length | 21.5 |
| Width | 10.9 |
| Depth | 4 |
| **IVPP V6027-3** |  |
| **Left scapula and coracoid** |  |
| ***Scapula*** |  |
| Proximodistal (=dorsoventral) height of scapula | 93.3 |
| Anteroposterior length of scapula proximally | 28.5 |
| Anteroposterior length of scapula proximally at proximalmost anteroposteriorly complete part of blade | 23.8 |
| Anteroposterior length of scapula distally | 41.1 |
| Minimum anteroposterior length of scapula | 18.7 |
| Distance from proximal end of shaft to point of minimum anteroposterior length | 39.2 |
| Mediolateral thickness of scapula proximally | 16.9 |
| Mediolateral thickness of scapula distally | 5.8 |
| Mediolateral width of glenoid area of scapula | 16.5 |
| Dorsoventral height of glenoid area of scapula | 8.3 |
| Mediolateral width of m. triceps brachii tuber | 5.7 |
| Dorsoventral height of m. triceps brachii tuber | 5 |
| Distance that m. triceps brachii tuber projects from shaft | 2.2 |
| ***Coracoid*** |  |
| Anteroposterior length of preserved coracoid fragment | 38.3 |
| Mediolateral thickness of preserved coracoid fragment | 39.1 |
| Dorsoventral height of preserved coracoid fragment | 13.7 |
| Length of coracoid foramen | 4 |
| Height of coracoid foramen | 3.5 |
| Width of glenoid area of coracoid | 23.9 |
| Height of glenoid area of coracoid | 14.1 |
| **IVPP V6027-4** |  |
| **Right scapula and coracoid** |  |
| ***Scapula*** |  |
| Proximodistal (=dorsoventral) height of scapula | 102.2 |
| Anteroposterior length of scapula proximally | 50 |
| Anteroposterior length of scapula distally | 42 |
| Minimum anteroposterior length of scapula | 18.1 |
| Distance from proximal end of shaft to point of minimum anteroposterior length | 44.1 |
| Mediolateral thickness of scapula proximally | 17.2 |
| Mediolateral thickness of scapula distally | 7.2 |
| Mediolateral thickness of glenoid area of scapula | 16.1 |
| Dorsoventral height of glenoid area of scapula | 12.4 |
| Mediolateral thickness of m. triceps brachii tuber | 5.5 |
| Dorsoventral height of m. triceps brachii tuber | 7.2 |
| Degree of projection of m. triceps brachii tuber from shaft | 2.2 |
| ***Coracoid*** |  |
| Anteroposterior length of preserved coracoid fragment | 24.8 |
| Mediolateral thickness of preserved coracoid fragment | 9.3 |
| Dorsoventral height of preserved coracoid fragment | 11.8 |
| Length of coracoid foramen | 3.6 |
| Height of coracoid foramen | 3.8 |
| **IVPP V6027-5** |  |
| **Right humerus** |  |
| Length | 93.5 |
| Width of distal end | 28.9 |
| Width of proximal end | 29.7 |
| Dorsoventral height of distal end | 10.5 |
| Dorsoventral height of proximal end | 15.7 |
| Distance from distal end of deltopectoral crest to distal end of humerus | 50.9 |
| Minimum diameter at midshaft in any direction | 8.84 |
| Circumference at midshaft | 35 |
| Minimum diameter of shaft in any direction | 8.2 |
| Distance to point of minimum diameter of shaft from distal end | 35 |
| Minimum circumference of shaft | 33 |
| Distance to point of minimum circumference of shaft from distal end | 52.1 |
| Minimum mediolateral diameter of shaft | 10.8 |
| Point of minimum mediolateral diameter of shaft from distal end | 43.5 |
| Minimum dorsoventral diameter of shaft | 8.2 |
| Point of minimum dorsoventral diameter of shaft from distal end | 33 |
| **IVPP V6027-6** |  |
| **Right ulna** |  |
| Proximodistal length of ulna | 88 |
| Mediolateral width at proximal end | 20.1 |
| Dorsoventral depth at proximal end | 13.6 |
| Mediolateral width at distal end | 12.1 |
| Dorsoventral depth at distal end | 6.5 |
| Mediolateral width at midshaft | 8 |
| Dorsoventral depth at midshaft | 5.1 |
| Minimum diameter in any direction | 4.7 |
| Proximodistal length from distal end to point of minimum diameter | 43.1 |
| Circumference at midshaft | 24 |
| Minimum circumference | 20 |
| Proximodistal length from distal end to point of minimum circumference | 24 |
| Minimum diameter of shaft in dorsal/ventral view | 4.6 |
| Point of minimum mediolateral diameter of shaft from distal end | 38.2 |
| Minimum diameter of shaft in medial/lateral view | 6.4 |
| Point of minimum dorsoventral diameter of shaft from distal end | 27.3 |
| **IVPP V6027-7** |  |
| **Right radius** |  |
| Proximodistal length | 76 |
| Mediolateral width at proximal end | 13.4 |
| Dorsoventral depth at proximal end | 8.3 |
| Mediolateral width at distal end | 10.8 |
| Dorsoventral depth at distal end | 8.9 |
| Mediolateral width at midshaft | 5.7 |
| Dorsoventral depth at midshaft | 5.3 |
| Minimum diameter in any direction | 5.3 |
| Proximodistal length from distal end to point of minimum diameter | 38 |
| Circumference at midshaft | 20 |
| Min. circumference | 20 |
| Proximodistal length from distal end to point of minimum circumference | 38 |
| Minimum diameter of shaft in dorsal/ventral view | 5.5 |
| Point of minimum mediolateral diameter of shaft from distal end | 31.2 |
| Minimum diameter of shaft in medial/lateral view | 5.7 |
| Point of minimum dorsoventral diameter of shaft from distal end | 45.8 |
| **IVPP V6027-8** |  |
| **Left cervical rib** |  |
| Length from proximalmost tip of flange to distal tip | 38.3 a.p. |
| Length from proximalmost tip of tuberculum to distal tip | 36.3 a.p. |
| Width from posterior margin of tuberculum to anterior margin of flange | 20.6 |
| Maximum width of flange | 11.5 |
| Maximum dorsoventral depth of proximal end of flange | 3.1 |
| Anteroposterior width of tip of tuberculum | 5.3 |
| Dorsoventral height of tip of tuberculum | 6.8 |
| Proximodistal length of tuberculum | 4.6 |
| Anteroposterior width of tip of capitulum | 4.1 |
| Dorsoventral height of tip of capitulum | 4.2 |
| Proximodistal length of capitulum | 2.6 |
| Depth of rib from ventral margin of capitulum to dorsal margin of tuberculum | 11.9 |
| **IVPP V6027-9** |  |
| **Left median osteoderm** |  |
| Length | 25.6 |
| Width | 12.7 |
| Dorsoventral depth | 4.2 |
| Maximum distance between lateral margin and longitudinal keel | 6.1 |
| Maximum distance between medial margin and longitudinal keel | 4.7 |
| ***“Turfanosuchus shageduensis*”** |  |
| **IVPP V6028-1** |  |
| **Right mandible** |  |
| Length of ramus | 155 |
| Maximum depth of ramus (across surangular and articular) | 18.1 |
| Depth of anterior end of ramus | 10.5 |
| Depth of posterior end of ramus | 16.8 |
| Width of ramus at widest (just anterior to presumed position of angle) | 14.1 |
| Width of anterior end of ramus | 13.1 |
| Width of posterior end of ramus | 11.6 |
| ***Teeth*** |  |
| ***Tooth 1 (anteriormost identifiable preserved tooth)*** | |
| Buccolingual width | 2.1 |
| Apicobasal height | 2.4 a.p. |
| Mesiodistal length | 2.6 |
| ***Tooth 2*** |  |
| Buccolingual width | 2.2 |
| Apicobasal height | 1.3 a.p. |
| Mesiodistal length | 2.4 |
| ***Tooth 3*** |  |
| Buccolingual width | 2.5 |
| Apicobasal height | 2.4 a.p. |
| Mesiodistal length | 2.2 |
| ***Tooth 4*** |  |
| Buccolingual width | 1.8 |
| Apicobasal height | 1.4 a.p. |
| Mesiodistal length | 2.1 |
| ***Tooth 5*** |  |
| Buccolingual width | 2.1 |
| Apicobasal height | 1.7 a.p. |
| Mesiodistal length | 2.5 |
| ***Prearticular*** |  |
| Max. height (oblique) | 14.2 |
| Height at anterior end (oblique) | 8.9 |
| Max. height (directly dorsoventral) | 14 |
| Max. mediolateral width of posterior section | 4.4 |
| Maximum mediolateral width of narrow anterior laterally rounded section | 5 |
| Max. height of anterior rounded strip | 6.4 |
| Max. length of anterior rounded strip | 23.2 |
| Max. length of posterior section (directly anteroposteriorly) | 20.4 |
| Max. length of posterior section (oblique) | 21.4 |
| ***Splenial*** |  |
| Max. height | 10.4 |
| **IVPP V6028-2** |  |
| **Cervical vertebrae** |  |
| ***Anteriormost cervical vertebra preserved*** |  |
| Length of centrum | 21.8 est. |
| Height of centrum anteriorly | 10.6 a.p. |
| Height of centrum posteriorly | 12.2 |
| Width of centrum anteriorly excl. apophyses | 14.3 |
| Width of centrum posteriorly excl. apophyses | 14 |
| Width of lower part of centrum at narrowest | 6.5 |
| Height of centrum at shortest | 8 |
| Width of right diapophysis at base | 6.9 |
| Height of right diapophysis at base | 2.6 |
| Width of right diapophysis at tip | 6.3 est. |
| Height of right diapophysis at tip | 2.9 est. |
| Width of left diapophysis at base | 5.7 |
| Height of left diapophysis at base | 3.9 |
| Width of left diapophysis at tip | 3.5 a.p. |
| Height of left diapophysis at tip | 3.4 a.p. |
| ***Second from anteriormost cervical vertebra preserved*** |  |
| Length of centrum | 19.3 |
| Height of centrum anteriorly | 12.5 |
| Height of centrum posteriorly | 12.4 |
| Width of centrum anteriorly excl. apophyses | 12.7 |
| Width of centrum posteriorly excl. apophyses | 13.9 |
| Width of lower part of centrum at narrowest | 7.6 |
| Height of centrum at shortest | 8.5 |
| Width of right diapophysis at base | 7.1 |
| Height of right diapophysis at base | 4.7 |
| Width of right diapophysis at tip | 6.4 |
| Height of right diapophysis at tip | 4.9 |
| Width of right parapophysis | 4.9 |
| Height of right parapophysis | 4.1 |
| Width of left diapophysis at base | 7.8 |
| Height of left diapophysis at base | 5.7 |
| Width of left diapophysis at tip | 7 |
| Height of left diapophysis at tip | 5.6 |
| Width of left parapophysis | 4.6 |
| Height of left parapophysis | 4.3 |
| ***Third from anteriormost cervical vertebra preserved*** |  |
| Length of centrum | 17.1 |
| Height of centrum anteriorly | 13.5 |
| Height of centrum posteriorly | 12.2 |
| Width of centrum anteriorly excl. apophyses | 13.1 |
| Width of centrum posteriorly excl. apophyses | 14.6 |
| Width of lower part of centrum at narrowest | 7.25 |
| Height of centrum at shortest | 8.6 |
| Width of right diapophysis at base | 8.7 |
| Height of right diapophysis at base | 4.3 |
| Width of right diapophysis at tip | 6.5 |
| Height of right diapophysis at tip | 3.4 |
| Width of right parapophysis | 4.4 |
| Height of right parapophysis | 4.9 |
| Width of left diapophysis at base | 9.2 |
| Height of left diapophysis at base | 3.5 |
| Width of left diapophysis at tip | 6.5 |
| Height of left diapophysis at tip | 3.4 |
| Width of left parapophysis | 5.2 |
| Height of left parapophysis | 3.4 |
| ***Fourth from anteriormost cervical vertebra preserved*** |  |
| Length of centrum | 19.9 |
| Height of centrum anteriorly | 12.9 |
| Height of centrum posteriorly | 12.4 |
| Width of centrum anteriorly excl. apophyses | 15.8 |
| Width of centrum posteriorly excl. apophyses | 15.2 |
| Width of lower part of centrum at narrowest | 7.6 |
| Height of centrum at shortest | 8.3 |
| Width of right diapophysis at base | 8.3 |
| Height of right diapophysis at base | 3.8 |
| Width of right diapophysis at tip | 6.3 |
| Height of right diapophysis at tip | 3.3 |
| Width of right parapophysis | 5.2 |
| Height of right parapophysis | 2.9 |
| Width of left diapophysis at base | 7.1 |
| Height of left diapophysis at base | 4.1 |
| Width of left diapophysis at tip | 7.6 |
| Height of left diapophysis at tip | 3.5 |
| Width of left parapophysis | 4.7 |
| Height of left parapophysis | 3.4 |
| ***Fifth from anteriormost cervical vertebra preserved*** |  |
| Length of centrum | 19 |
| Height of centrum anteriorly | 13.1 |
| Height of centrum posteriorly | 14.1 |
| Width of centrum anteriorly excl. apophyses | 16.3 |
| Width of centrum posteriorly excl. apophyses | 14.1 a.p. |
| Width of lower part of centrum at narrowest | 6.5 |
| Height of centrum at shortest | 7.3 a.p. |
| Width of right parapophysis | 5.8 |
| Height of right parapophysis | 4.7 |
| Width of left parapophysis | 6.6 |
| Height of left parapophysis | 6.2 |
| ***Sixth from anteriormost cervical vertebra preserved*** |  |
| Length of centrum | 18.2 a.p. |
| Height of centrum anteriorly | 12.9 |
| Height of centrum posteriorly | 8.8 a.p. |
| Width of centrum anteriorly excl. apophyses | 15 |
| Width of centrum posteriorly excl. apophyses | 12.4 a.p. |
| Width of lower part of centrum at narrowest | 7.4 |
| Height of centrum at shortest | 7.5 |
| Width of right parapophysis | 7.7 |
| Height of right parapophysis | 7 |
| Width of left parapophysis | 7.9 |
| Height of left parapophysis | 7.3 |
| **IVPP V6028-3** |  |
| **Right scapula and partial coracoid** |  |
| ***Scapula*** |  |
| Proximodistal of scapula | 93.2 a.p. |
| Anteroposterior length of scapula proximally | 34.4 a.p. |
| Anteroposterior length of scapula distally | 47.4 a.p. |
| Minimum anteroposterior length of scapula | 10.5 a.p. |
| Distance from proximal end of shaft to point of minimum anteroposterior length | 19.7 a.p. |
| Mediolateral thickness of scapula proximally | 13.4 |
| Mediolateral thickness of scapula distally | 6.3 |
| Mediolateral width of glenoid area of scapula | 14.8 |
| Dorsoventral height of glenoid area of scapula | 9.2 |
| ***Coracoid*** |  |
| Anteroposterior length of preserved coracoid fragment | 33.5 |
| Mediolateral thickness of preserved coracoid fragment | 10.4 |
| Dorsoventral height of preserved coracoid fragment | 31.3 |
| Width of glenoid area of coracoid | 11 |
| Height of glenoid area of coracoid | 16.1 |
| **IVPP V6028-4** |  |
| **Right humerus** |  |
| Length | 84.1 |
| Width of distal end | 27.2 |
| Width of proximal end | 35.8 |
| Depth of distal end perpendicular to long axis | 12 |
| Depth of proximal end perpendicular to long axis | 12.2 |
| Distance from proximal end of humerus to distal end of deltopectoral crest | 27.9 |
| Mediolateral diameter at midshaft | 9.3 |
| Dorsoventral diameter at midshaft | 7.6 |
| Circumference at midshaft | 28 |
| Minimum diameter of shaft in any direction | 7.4 |
| Distance from distal end to point of minimum shaft diameter | 43.7 |
| Minimum circumference of shaft | 28 |
| Distance from distal end to point of minimum shaft circumference | 42 |
| Minimum mediolateral diameter of shaft | 9.3 |
| Point of minimum mediolateral diameter of shaft in from proximal end | 48.2 |
| Minimum diameter of shaft in medial/lateral view | 6.2 |
| Point of minimum dorsoventral diameter of shaft from proximal end | 31.3 |
| **IVPP V6028-5** |  |
| **Right femur** |  |
| Length | 127 |
| Width of distal end along long axis (across condyles) | 23 |
| Width of proximal end along long axis | 30 |
| Depth of distal end perpendicular to long axis | 21.1 |
| Depth of proximal end perpendicular to long axis | 13.5 |
| Proximodistal length of fourth trochanter | 25.7 |
| Mediolateral max. width of fourth trochanter | 7.5 |
| Dorsoventral height of fourth trochanter | 4.4 |
| Dorsoventral depth of lateral condyle (incl. crista tibiofibularis) | 19.3 a.p. |
| Mediolateral width of lateral condyle | 13.7 |
| Dorsoventral depth of medial condyle | 16.7 |
| Mediolateral width of medial condyle | 9.6 a.p. |
| Diameter at midshaft in dorsal view | 16 |
| Diameter at midshaft in lateral view | 10.3 |
| Circumference at midshaft | 44 |
| Minimum diameter of shaft in any direction | 9.4 |
| Distance of point of minimum diameter of shaft from proximal end | 47.2 |
| Minimum circumference of shaft | 44 |
| Distance of point of minimum circumference of shaft from proximal end | 65 |
| Minimum diameter of shaft in dorsal/ventral view | 14.9 |
| Point of minimum mediolateral diameter of shaft from proximal end | 85 |
| Minimum diameter of shaft in medial/lateral view | 9.7 |
| Point of minimum dorsoventral diameter of shaft from proximal end | 59.2 |
| **IVPP V6028-6** |  |
| **Right tibia** |  |
| Length | 104.2 |
| Anteroposterior width of distal end | 13.2 a.p. |
| Anteroposterior width of proximal end | 23 a.p. |
| Width of distal end mediolaterally | 14.7 a.p. |
| Width of proximal end mediolaterally | 21.5 a.p. |
| Diameter at midshaft in dorsal view | 8.1 |
| Diameter at midshaft in lateral view | 11.8 |
| Circumference at midshaft | 34 |
| Minimum diameter of shaft in any direction | 6.8 |
| Distance of point of minimum diameter of shaft from proximal end | 39.7 |
| Minimum circumference of shaft | 34 |
| Distance of point of minimum circumference of shaft from proximal end | 52 |
| Minimum diameter of shaft in dorsal/ventral view | 6.8 |
| Point of minimum mediolateral diameter of shaft from proximal end | 41.3 |
| Minimum diameter of shaft in medial/lateral view | 12.3 |
| Point of minimum dorsoventral diameter of shaft from proximal end | 52 |
| Max. width of shaft where broken | 12.6 |
| Width of lateral (wider) bone wall along same axis as max. width of shaft | 2.8 |
| Width of medial (narrower) bone wall along same axis as max. width of shaft | 2.5 |
| Width of shaft between bone walls along same axis as max. width of shaft | 6.3 |
| Distance from break to distal end | 30.7 |
| **IVPP V6028-7-9** |  |
| ***Right ulna*** |  |
| Length | 84 a.p. |
| Width of distal end along long axis | 11.6 |
| Width of proximal end along long axis | 19.6 |
| Depth of distal end perpendicular to long axis | 5.5 |
| Depth of proximal end perpendicular to long axis | 10.6 |
| Diameter at midshaft in dorsal view | 4.9 a.p. |
| Diameter at midshaft in lateral view | 8.3 a.p. |
| Circumference at midshaft | 22 |
| Minimum diameter of shaft in any direction | 4.2 |
| Distance of point of minimum diameter of shaft from proximal end | 38.4 |
| Minimum circumference of shaft | 22 a.p. |
| Distance of point of minimum circumference of shaft from proximal end | 64.1 |
| Minimum diameter of shaft in dorsal/ventral view | 4 |
| Point of minimum mediolateral diameter of shaft from proximal end | 38.4 |
| Minimum diameter of shaft in medial/lateral view | 7.5 |
| Point of minimum dorsoventral diameter of shaft from proximal end | 54.7 |
| ***Right radius*** |  |
| Length | 74.4 |
| Width of distal end along long axis | 7.5 |
| Width of proximal end along long axis | 12.4 |
| Depth of distal end perpendicular to long axis | 7.1 |
| Depth of proximal end perpendicular to long axis | 7.4 a.p. |
| Diameter at midshaft in dorsal view | 6.3 a.p. |
| Diameter at midshaft in lateral view | 6.3 |
| Circumference at midshaft | 24 |
| Minimum diameter of shaft in any direction | 5.3 |
| Distance of point of minimum diameter of shaft from proximal end | 52.2 |
| Minimum circumference of shaft | 21 a.p. |
| Distance of point of minimum circumference of shaft from proximal end | 46.5 |
| Minimum diameter of shaft in dorsal/ventral view | 6 |
| Point of minimum mediolateral diameter of from proximal end | 49.1 |
| Minimum diameter of shaft in medial/lateral view | 5.3 |
| Point of minimum dorsoventral diameter of shaft from proximal end | 52.2 |
| ***Fibula*** |  |
| Length | 88.1 a.p. |
| Width of distal end along long axis | 14.6 |
| Width of proximal end along long axis | 8.9 a.p. |
| Depth of distal end perpendicular to long axis | 5.5 |
| Depth of proximal end perpendicular to long axis | 3.8 a.p. |
| Diameter at midshaft in dorsal view | 4.5 |
| Diameter at midshaft in lateral view | 7 |
| Circumference at midshaft | 22 |
| Minimum diameter of shaft in any direction | 3.8 |
| Distance of point of minimum diameter of shaft from proximal end | 60 |
| Minimum circumference of shaft | 19 |
| Distance of point of minimum circumference of shaft from proximal end | 60 |
| Minimum diameter of shaft in dorsal/ventral view | 3.9 |
| Point of minimum mediolateral diameter of shaft from proximal end | 60 |
| Minimum diameter of shaft in medial/lateral view | 7.3 |
| Point of minimum dorsoventral diameter of from proximal end | 56 |
| Max. width of shaft where broken | 4.5 |
| Width of wider bone wall along same axis as max. width of shaft | 1.5 |
| Width of narrower bone wall along same axis as max. width of shaft | 1.3 |
| Width of centre of shaft along same axis as max. width of shaft | 1.7 |
| Point of break from distal end | 39.5 |
| **"*Wangisuchus tzeyii*"** |  |
| **IVPP V2701-1** |  |
| **Partial left maxilla** |  |
| Length | 93.3 |
| Maximum dorsoventral depth | 35.6 |
| Depth at posterior end | 19.9 |
| Depth at anterior end | 24.2 |
| Minimum dorsoventral depth | 20.6 |
| Maximum width | 12.9 |
| Width at anterior end | 6.9 |
| Width at posterior end | 8 |
| Minimum width | 6.3 |
| Number of teeth | 3 |
| Number of alveoli | 12 |
| ***Anteriormost preserved root*** |  |
| Mediolateral width | 2 |
| Dorsoventral height | 6.7 |
| Anteroposterior length | 6.1 |
| ***Anteriormost preserved alveolus*** |  |
| Mediolateral width | 3.6 a.p. |
| Dorsoventral height | 4 a.p. |
| Anteroposterior length | 2.2 a.p. |
| ***Second from anteriormost preserved interdental plate*** |  |
| Mediolateral width |  |
| Dorsoventral height | 8.6 |
| Anteroposterior length | 2.9 |
| ***Second from anteriormost preserved root*** |  |
| Dorsoventral height | 9.1 |
| Anteroposterior length | 4.8 |
| ***Second from anteriormost preserved alveolus*** |  |
| Mediolateral width | 3 |
| Dorsoventral height | 3.6 |
| Anteroposterior length | 4.9 |
| ***Third from anteriormost preserved interdental plate*** |  |
| Dorsoventral height | 11.1 |
| Anteroposterior length | 5.4 |
| ***Third from anteriormost preserved root*** |  |
| Dorsoventral height | 11.4 |
| Anteroposterior length | 4 |
| ***Third from anteriormost preserved alveolus*** |  |
| Mediolateral width | 4.1 |
| Dorsoventral height | 3.9 |
| Anteroposterior length | 4.8 |
| ***Fourth from anteriormost preserved interdental plate*** |  |
| Dorsoventral height | 12.7 |
| Anteroposterior length | 5.6 |
| ***Fourth from anteriormost preserved root*** |  |
| Dorsoventral height | 14.9 |
| Anteroposterior length | 5.5 |
| ***Fourth from anteriormost preserved alveolus*** |  |
| Mediolateral width | 4 |
| Dorsoventral height | 3.4 |
| Anteroposterior length | 5 |
| ***Fifth from anteriormost preserved interdental plate*** |  |
| Dorsoventral height | 16.5 |
| Anteroposterior length | 6 |
| ***Fifth from anteriormost preserved root*** |  |
| Dorsoventral height | 15.3 |
| Anteroposterior length | 3.9 |
| ***Fifth from anteriormost preserved alveolus*** |  |
| Mediolateral width | 4.3 |
| Dorsoventral height | 3.5 |
| Anteroposterior length | 7 |
| ***Sixth from anteriormost preserved interdental plate*** |  |
| Dorsoventral height | 15.2 |
| Anteroposterior length | 6.7 |
| ***Sixth from anteriormost preserved root*** |  |
| Dorsoventral height | 12.4 |
| Anteroposterior length | 5.4 |
| ***Sixth from anteriormost preserved alveolus*** |  |
| Mediolateral width | 3.4 |
| Dorsoventral height | 5.5 |
| Anteroposterior length | 6.3 |
| ***Seventh from anteriormost preserved interdental plate*** |  |
| Dorsoventral height | 10.2 |
| Anteroposterior length | 9.1 |
| ***Seventh from anteriormost preserved root*** |  |
| Dorsoventral height | 6.7 |
| Anteroposterior length | 4.9 |
| ***Seventh from anteriormost preserved alveolus*** |  |
| Mediolateral width | 2.7 |
| Dorsoventral height | 11.1 |
| Anteroposterior length | 7.3 |
| ***Eighth from anteriormost preserved tooth*** |  |
| Mediolateral width | 2.6 |
| Dorsoventral height | 5.1 |
| Anteroposterior length | 4.7 |
| ***Eighth from anteriormost preserved interdental plate*** |  |
| Dorsoventral height | 9 |
| Anteroposterior length | 4.9 |
| ***Eighth from anteriormost preserved root*** |  |
| Dorsoventral height | 4.6 |
| Anteroposterior length | 4.7 |
| ***Eighth from anteriormost preserved alveolus*** |  |
| Mediolateral width | 3 |
| Dorsoventral height | 9.5 |
| Anteroposterior length | 8 |
| ***Ninth from anteriormost preserved root*** |  |
| Mediolateral width | 3.7 est. |
| Dorsoventral height | 9.6 est. |
| Anteroposterior length | 5.2 |
| ***Ninth from anteriormost preserved alveolus*** |  |
| Mediolateral width | 4.6 |
| Anteroposterior length | 5 est. |
| ***Tenth from anteriormost preserved tooth*** |  |
| Mediolateral width | 3.3 |
| Dorsoventral height | 4 |
| Anteroposterior length | 4.9 |
| ***Tenth from anteriormost preserved root*** |  |
| Dorsoventral height | 7.8 |
| Anteroposterior length | 4.2 |
| ***Tenth from anteriormost preserved alveolus*** |  |
| Mediolateral width | 3.9 est. |
| Dorsoventral height | 2.1 |
| Anteroposterior length | 4.6 |
| **IVPP V2702** |  |
| **?Right ?maxilla** |  |
| Length | 32.9 |
| Width | 8.5 |
| Height | 21 |
| **IVPP V2703** |  |
| **Right maxilla** |  |
| Length | 54.2 |
| Maximum depth | 34.9 |
| Depth at posterior end | 16.5 |
| Depth at anterior end | 15.8 |
| Minimum depth | 16.1 |
| Maximum width | 9.8 |
| Width at anterior end | 5.7 |
| Width at posterior end | 6 |
| Min. width | 5.9 |
| Number of teeth | 0 |
| Number of alveoli | 6 |
| **IVPP V2704** |  |
| **Right maxilla** |  |
| Length | 65.8 |
| Max. depth | 25.3 |
| Depth at posterior end | 14.2 |
| Depth at anterior end | 14.2 |
| Minimum depth | 14.2 |
| Maximum width | 8.2 |
| Width at anterior end | 3.9 |
| Width at posterior end | 5.6 |
| Minimum width | 5.6 |
| Number of teeth | 3 |
| Number of alveoli | 9 |
