# Supplementary material for: Systematics of putative euparkeriids (Diapsida: Archosauriformes) from the Triassic of China
Source: PeerJ. 2014 Nov 25;2:e658. doi: 10.7717/peerj.658 (PMC4250070; doi:10.7717/peerj.658)
Supplement: Table S2 — Names of localities, towns, administrative divisions and formations used in this article, showing their equivalents in simplified Chinese, Pinyin, and previously published romanizations and translations. [file peerj-02-658-s002.pdf]

Table S2. **Supplementary table of Chinese names.** Names of localities, towns, administrative divisions and formations used in this article, showing their equivalents in simplified Chinese, Pinyin, and previously published romanizations and translations.

Name used in this paper	Name in simplified Chinese characters	Name in Pinyin	Other romanizations/translations used in previous palaeontological publications
Autonomous Region	自治区	Zìzhìqū	
Banner	旗	Qí	
County	县	Xiàn	
Ermaying	二马营	Èrmǎyíng	Er-Ma-Ying (Wu 1982)
Fugu	府谷	Fǔgǔ	
Heshanggou			
Jungar	准格尔	Zhǔngé'ěr	Zhun-Ge-Er (“Zhun-Ge-Er-Qi”; Wu 1982)
Louzeyu	楼则峪	Lóuzéyù	Lotzeyue (Young 1964)
Nei Mongol	内蒙古	Nèi Měnggǔ	Inner Mongolia (Wu 1982)
Province	省	Shěng	
Shaanxi	陕西	Shǎnxī	Shansi (Wu 1982)
Shanxi	山西	Shānxī	
Wuxiang	武乡	Wǔxiāng	Wuhsiang (Young 1964)
Xishiwa	西什凹	Xīshíwā	Shishihwa (Young 1964)
